# Resurgence of Zoonotic Highly Pathogenic Avian Influenza A(H5N1) in Cambodia

**DOI:** 10.1056/NEJMc2504302

**Published:** 2025-10-23

**Authors:** Jurre Y. Siegers, Ruopeng Xie, Kimberly M. Edwards, Alexander M.P. Byrne, Shu Hu, Ruixuan Wang, Sokhoun Yann, Sarath Sin, Songha Tok, Kimlay Chea, Sreyviseth Horm, Chenthearath Rith, Seangmai Keo, Leakhena Pum, Veasna Duong, Heidi Auerswald, Yisuong Phou, Sonita Kol, Andre Spiegel, Ruth Harvey, Sothyra Tum, San Sorn, Bunnary Seng, Yi Sengdoeurn, Chau Darapheak, Chin Savuth, Makara Hak, Vanra Ieng, Sarika Patel, Peter Thielen, Filip F. Claes, Nicola S. Lewis, Ly Sovann, Erik A. Karlsson, Vijaykrishna Dhanasekaran

**Affiliations:** 1 Institut Pasteur du Cambodge, Phnom Penh, Cambodia; 2 The University of Hong Kong, Hong Kong SAR, China; 3 The Francis Crick Institute, London; 4 National Animal Health and Production Research Institute, Phnom Penh, Cambodia; 5 Ministry of Health, Phnom Penh, Cambodia; 6 National Institute of Public Health, Ministry of Health, Phnom Penh, Cambodia; 7 Food and Agriculture Organization of the United Nations (FAO) Country Office, Phnom Penh, Cambodia; 8 World Health Organization Country Office, Phnom Penh, Cambodia; 9 Johns Hopkins University Applied Physics Laboratory, Laurel, Maryland; 10 FAO Emergency Centre for Transboundary Animal Diseases, Bangkok, Thailand

## To the Editor:

After a decade of no reported human cases, Cambodia faces a resurgence of highly pathogenic avian influenza (HPAI) H5N1 with an overall 38% fatality rate. Cases occur primarily in children and adolescents exposed to infected poultry (Figure, Table S1). Between February 2023 and August 2024, sixteen infections were detected through Cambodia’s long-standing influenza-like-illness (ILI) and severe acute respiratory infection (SARI) surveillance systems, which were strengthened by expanded laboratory testing capacity developed during the COVID-19 pandemic. All patients reported exposure to sick or dead poultry, and outbreak investigations identified contemporary, genetically similar viruses in poultry collected in or around case households or from active, longitudinal live bird market surveillance. Genomic sequencing revealed avian origin for all human infections, and sequences have been publicly shared via the Global Initiative on Sharing All Influenza Data (GISAID).

While most cases occurred in separate households, four households experienced multiple infections. A father-daughter cluster initially suggested person-to-person transmission, but outbreak investigations by the Ministry of Health and WHO revealed simultaneous symptom onset and direct contact with infected backyard poultry^1^, highlighting poultry exposure as the likely driver of these infections.

Initial cases in February 2023 (Figure, blue shading) were caused by the regionally endemic H5N1 clade 2.3.2.1e (previously classified as 2.3.2.1c under WHO nomenclature^2^ (ref)), whereas subsequent cases were associated with a novel reassortant virus. The local clade 2.3.2.1e acquired genes from clade 2.3.4.4b and low-pathogenicity avian influenza viruses, likely via wild birds or undetected poultry transmission, though limited genomic data precludes definitive source attribution (Figure, Figure S1). This reassortant has spread across the Greater Mekong Subregion, replacing earlier strains. Notably, this virus carries genomic signatures (e.g., PB2:E627K) linked to enhanced polymerase activity, virulence, and replication capacity in birds and mammals^3^, posing increased potential risk to the poultry industry as well as for zoonotic transmission potential (Table S2).

This novel reassortment, genotype replacement, and resurgence in humans underscores the dynamic and unpredictable nature of HPAI H5N1 virus evolution, particularly in regions with dense poultry-human interfaces. The genetic landscape of HPAI is rapidly shifting. Since 2021, HPAI H5N1 viruses have expanded in host and geographic range, heightening the risk of zoonotic spillover^4,5^. Cambodia’s outbreak highlights the need for One Health investments: integrating real-time surveillance, cross-sectoral data sharing, and genomic monitoring to mitigate pandemic risks.

## Supplementary Material

Supplementary Appendix

## Figures and Tables

**Figure.**
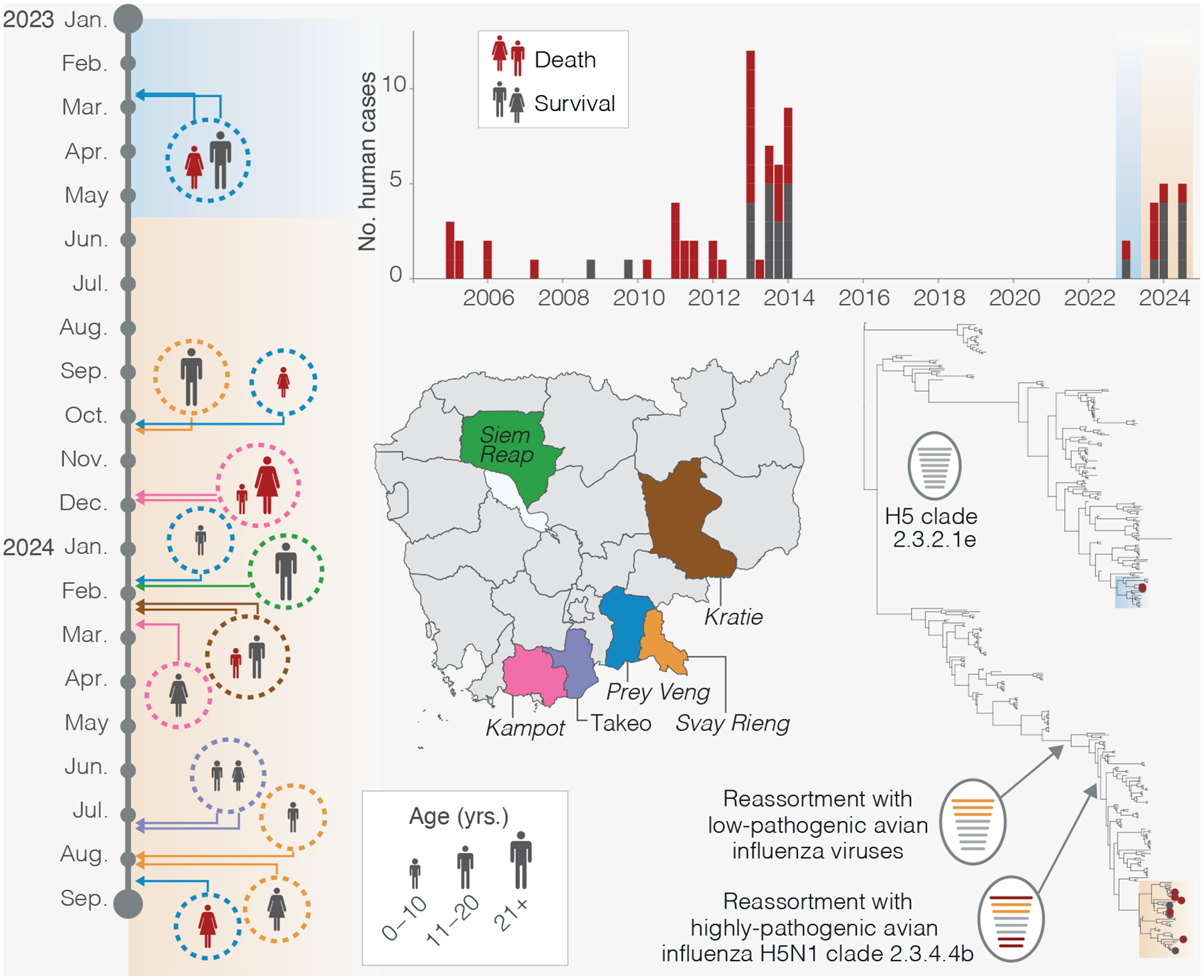
Human cases of highly pathogenic avian influenza in Cambodia. Timeline (left) details outbreak clusters (circles) by province (color-coded, see map) from February 2023–August 2024. Case silhouettes represent age, sex, and clinical outcome (gray: survival, red: fatal). Cases caused by historical 2.3.2.1e viruses are shaded in blue, novel reassortant cases are shaded in orange. Bar chart (top right) shows quarterly human case counts since 2005. Time-resolved phylogenetic tree (bottom right) reflects evolution of H5 clade 2.3.2.1e in poultry in southeast Asia since 2016. Terminal nodes (circles) denote human cases. Arrows highlight two distinct reassortant genotypes derived from clades 2.3.2.1e, 2.3.4.4b, and low-pathogenic avian influenza viruses. Virus diagrams represent influenza gene segments (PB2, PB1, PA, HA, NP, NA, M, and NS), ordered by length.
