# Supplementary material for: Resurgence of Zoonotic Highly Pathogenic Avian Influenza A(H5N1) in Cambodia
Source: N Engl J Med. Author manuscript; Available in PMC 2025 Nov 13. (PMC12614772; doi:10.1056/NEJMc2504302)
Supplement: Supplementary Appendix [file NIHMS2113651-supplement-Supplementary_Appendix.pdf]

Supplementary Appendix

Table of Contents

Supplementary Table 1 2

Supplementary Table 2 3

Supplementary Figure 1 4

Supplementary Figure 2 5

Supplementary Data 1 6

Ethics Statement 6

Acknowledgments 6

Funding Statement 6

Author Contributions 7

References 7

Supplementary Table 1

Supplementary Table 1. Human cases of highly pathogenic avian influenza H5N1 clade 2.3.2.1e virus infection.

Date of sampling	*Province*	*Age (yrs.)*	*Sex*	*Case ascertainment method**	*Clinical outcome*	*GISAID sequence accession no* [#]
21-Feb-2023	Prey Veng	11	F	ILI/SARI	Deceased	EPI_ISL_17024123
23-Feb-2023	Prey Veng	49	M	Father of above case	Recovered	EPI_ISL_17069010
6-Oct-2023	Prey Veng	2	F	ILI/SARI	Deceased	EPI_ISL_18373263
7-Oct-2023	Svay Rieng	50	M	ILI/SARI	Deceased	EPI_ISL_18366401
23-Nov-2023	Kampot	21	F	ILI/SARI	Deceased	EPI_ISL_18540514
24-Nov-2023	Kampot	4	F	Neighbor of above case	Recovered	EPI_ISL_18543643
23-Jan-2024	Prey Veng	3	M	ILI/SARI	Recovered	EPI_ISL_18823967
24-Jan-2024	Siem Reap	69	M	ILI/SARI	Recovered	EPI_ISL_18972144
8-Feb-2024	Kratie	9	M	ILI/SARI	Deceased	EPI_ISL_18879683
10-Feb-2024	Kratie	16	M	Brother of above case	Recovered	EPI_ISL_19270607
21-Feb-2024	Kampot	17	F	ILI/SARI	Recovered	N/A
5-July-2024	Takeo	3	M	ILI/SARI	Recovered	N/A
7-July-2024	Takeo	5	F	Cousin of above case, same household	Recovered	N/A
30-July-2024	Svay Rieng	4	M	ILI/SARI	Recovered	EPI_ISL_19312043
02-Aug-2024	Svay Rieng	16	F	ILI/SARI	Recovered	EPI_ISL_19312044
17-Aug-2024	Prey Veng	15	F	ILI/SARI	Deceased	EPI_ISL_19353003

* ILI/SARI, influenza-like-illness or severe acute respiratory infection, identified through syndromic surveillance; close contacts identified by outbreak investigations were administered Tamiflu.

[#] Viruses sequenced in this study were submitted to the Global Initiative on Sharing All Influenza Data (https://gisaid.org); N/A, not available.

Supplementary Table 2

Supplementary Table 2. Amino acid markers of phenotypic significance detected in human H5N1 clade 2.3.2.1e virus sequences. Amino acid signatures associated with phenotypic effects, adapted from Suttie et al. (2019).

Protein	Amino acid position (H5 HA numbering)	Feb-2023	Oct-2023	Nov-2023	Jan-2024	Feb-2024	Aug-2024	Phenotypic effect
PB2	L89V, G309D	Yes	Yes	Yes	Yes	Yes	Yes	Increases polymerase activity and virulence in mammals
	L89V, G309D, T339K, R477G, I495V, K627E, A676T	No	No	No	Yes	Yes	No	
	E627K	E	K	K	K	E	K	
PB1	D3V	V	V	V	V	V	V	Increases polymerase activity and viral replication
	473V	V	V	V	V	V	V	
	D622G	G	G	G	G	G	D	Increases polymerase activity and virulence in mice
PA	N383D	D	D	D	D	D	D	Increases polymerase activity in mammalian and avian cell lines
HA	323 to 330 (R-X-R, K-R)	Yes	Yes	Yes	Yes	Yes	Yes	HPAI polybasic cleavage site
	S107R	R	R	R	R	R	R	Increases virulence in chickens and mice and the pH of fusion
	T108I	I	I	I	I	I	I	
	D94N	S	N	N	N	N	N	Increases α-2,6 receptor binding
	S154N	N	D	D	D/N	N	N	
	S155N	N	N	N	N	N	N	
	T156A	A	A	A	A	A	A	
	K189R	R	K	K	K	K	K/N	
	R497K	R	R	R	K	R	K	
NP	M105V	V	V	V	V	V	V	Increases virulence in chickens
	A184K	K	K	K	K	K	K	Increases replication in avian cells and virulence in chickens
NA	49-68 del.	Yes	Yes	Yes	Yes	Yes	Yes	Increases virulence in mice
M1	N30D	D	D	D	D	D	D	Increases virulence in mice
	T215A	A	A	A	A	A	A	
	I43M	M	M	M	M	M	M	Increases virulence in mice, chickens and ducks
NS1	P42S	S	S	S	S	S	S	Increases virulence and decreases the antiviral response in mice
	K55E	K	E	E	E	E	E	Enhances replication in mammalian cells and decreases the interferon response
	K66E	E	E	E	E	E	E	
	C138F	F	F	F	F	F	F	
	80-84 del.	Yes	No	No	No	No	No	Increases virulence in mice
	L103F	F	F	F	F	F	F	
	I106M	M	M	M	M	M	M	
	V149A	A	A	A	A	A	A	Increases virulence and decreases the interferon response in chickens

Supplementary Figure 1

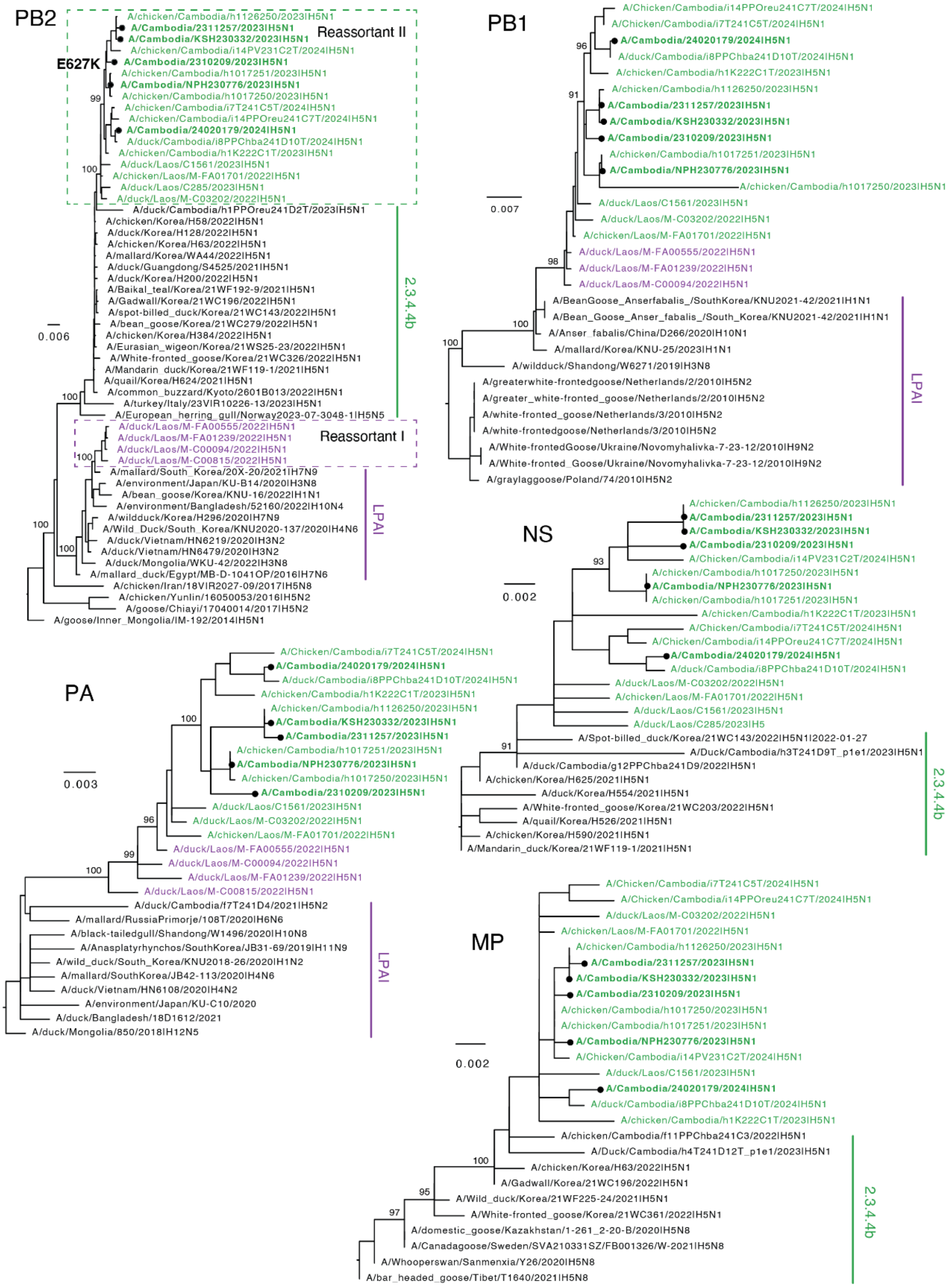

Supplementary Figure 1. Evolutionary origins of internal genes in H5N1 clade 2.3.2.1e reassortants. PB2, PB1, and PA genes were introduced from low-pathogenic avian influenza (LPAI) viruses (purple), followed by PB2, MP, and NS genes from highly pathogenic avian influenza (HPAI) H5N1 clade 2.3.4.4b viruses (green). The presented maximum likelihood trees subsampled from a large-scale analysis of BLAST-matched sequences. Bootstrap values are displayed at key nodes on the phylogenetic trunk. Black tips represent human cases.

Supplementary Figure 2

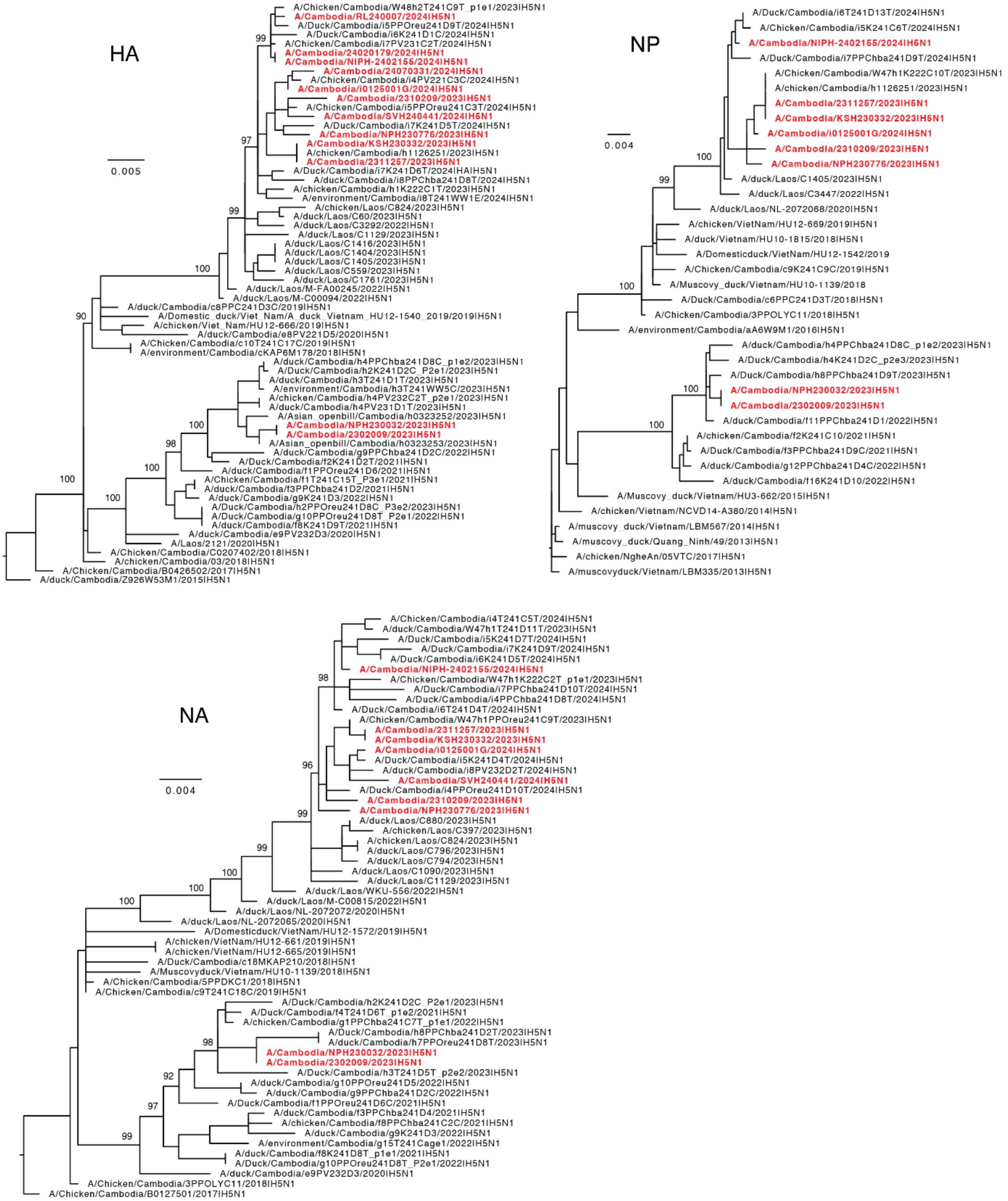

Supplementary Figure 2. Evolutionary relationships of HA, NA and NP genes. The presented maximum likelihood trees subsampled from a large-scale analysis of BLAST-matched sequences. Bootstrap values are displayed at key nodes on the phylogenetic trunk. Human cases are highlighted in red.

Supplementary Data 1
full list of GISAID acknowledgments are available in supplementary data 1.

Ethics Statement
All human H5 sequences and information (e.g. collection date, location) included in this analysis were obtained as part of human H5 case testing and One Health response as described below. All data included in this study is available in the public record. All patient samples were de-identified and no other individual-specific information was used in this study. Analyses in this study have been approved by the Cambodian National Ethics Committee for Health Research (#365NECHR/2024). IPC is a World Health Organization H5 Reference Laboratory and the Cambodian National Influenza Centre, with the necessary approvals and infrastructure for handling high pathogenicity avian influenza. No animal experimentation was performed in the context of this study.

Acknowledgments
The investigators thank everyone involved in the critical discussions and review of this manuscript. They also thank everyone involved in influenza surveillance and response in the Kingdom of Cambodia including teams at the National Institute for Public Health/National Public Health Laboratory, Cambodian Communicable Disease Control Department, Ministry of Health, National Animal Health and Production Institute, General Directorate of Animal Health and Production, Ministry of Agriculture, Forestry, and Fisheries, World Health Organization, and the Influenza Team in the Virology Unit at Institut Pasteur du Cambodge who contributed to this study. We gratefully acknowledge the authors from the originating laboratories responsible for obtaining the specimens and the submitting laboratories where genetic sequence data were generated and shared via the GISAID Initiative, on which this research is based (full list of GISAID acknowledgments are available in supplemental material). We also thank all NICs and laboratories that have supplied influenza viruses to the WHO Collaborating Centre for Reference and Research on Influenza for analysis. The text as published does not necessarily represent the official view of WHO or the FAO.

Funding Statement
Avian influenza work in the Virology Unit at Institut Pasteur du Cambodge was funded, in part, by the Food and Agriculture Organization of the United Nations, the World Health Organization, and the Bill and Melinda Gates Foundation awarded to E.A.K. H.A. is supported, in part, by the German Centre for International Migration and Development. P.M.T was supported by Johns Hopkins APL internal research and development. R.X., K.M.E., S.H., R.W., and V.D. are funded by the Research Grants Council of the Hong Kong Special Administrative Region, China, (Project Numbers T11-712/19-N, and PDFS2425-7S01), the U.S. National Institutes of Health (contract number 75N93021C00016), and Institut Pasteur (ACIP Project no 676-2023). The

funders had no role in study design, data collection and interpretation, or the decision to submit the work for publication.

Author Contributions

Conceptualization: J.Y.S., R.X., A.M.P.B., K.M.E., N.S.L., V. Dhanasekaran, and E.A.K. Data curation: J.Y.S., R.X., A.M.P.B., K.M.E., S.H., S.Y., S. Sin, S. Tok, K.C., S.V.H., C.R., S. Keo, L.P., H.A., Y.P., S. Kol, R.H., C.D., C.S., V.I., S.P., V. Dhanasekaran, and E.A.K. Formal analysis: J.Y.S., R.X., A.M.P.B., K.M.E., S.H., S.Y., S. Sin, S. Tok, K.C., S.V.H., C.R., S. Keo, L.P., H.A., Y.P., S. Kol, R.H., N.S.L., V. Dhanasekaran, and E.A.K. Funding acquisition: V. Duong, A.S., V.I., S.P., F.F.C., N.S.L., V. Dhanasekaran, and E.A.K. Investigation: J.Y.S., R.X., A.M.P.B., K.M.E., S.H., S.Y., S. Sin, S. Tok, K.C., S.V.H., C.R., S. Keo, L.P., Y.P., S. Kol, R.H., S. Tum, S. Sorn, B. Seng, Y.S., C.D., C.S., M.H., V.I., S.P., P.T., F.F.C., N.S.L., L.S., V. Dhanasekaran, and E.A.K. Methodology: J.Y.S., R.X., A.M.P.B., K.M.E., H.A., P.T., N.S.L., V. Dhanasekaran, and E.A.K. Project administration: V. Duong, H.A., A.S., S. Tum, S. Sorn, B. Seng, C.D., C.S., M.H., V.I., S.P., F.F.C., N.S.L., L.S., V. Dhanasekaran, and E.A.K. Resources: V. Duong, H.A., S. Tum, S. Sorn, B. Seng, C.D., C.S., M.H., V.I., S.P., P.T., F.F.C., N.S.L., L.S., V. Dhanasekaran, and E.A.K. Software: P.T. and V. Dhanasekaran Supervision: V. Duong, H.A., A.S., S. Tum, S. Sorn, B. Seng, Y.S., C.D., C.S., M.H., V.I., S.P., F.F.C., N.S.L., L.S., V. Dhanasekaran, and E.A.K. Validation: J.Y.S., R.X., A.M.P.B., K.M.E., S.Y., S.V.H., V. Dhanasekaran, and E.A.K. Visualization: J.Y.S., R.X., A.M.P.B., K.M.E., V. Dhanasekaran, and E.A.K. Writing - original draft: J.Y.S., R.X., A.M.P.B., K.M.E., P.T., F.F.C., N.S.L., V. Dhanasekaran, and E.A.K. Writing - review & editing: J.Y.S., R.X., A.M.P.B., K.M.E., S.H., S.Y., S. Sin, S. Tok, K.C., S.V.H., C.R., S. Keo, L.P., V. Duong, H.A., Y.P., S. Kol, A.S., R.H., S. Tum, S. Sorn, B. Seng, Y.S., C.D., C.S., M.H., V.I., S.P., P.T., F.F.C., N.S.L., L.S., V. Dhanasekaran, and E.A.K.

References

Suttie A, Deng YM, Greenhill AR, Dussart P, Horwood PF, Karlsson EA. Inventory of molecular markers affecting biological characteristics of avian influenza A viruses. Virus Genes 2019;55:739-68.
